# Genetic and phenotypic diversity of fecal *Candida albicans* strains in irritable bowel syndrome

**DOI:** 10.1038/s41598-022-09436-x

**Published:** 2022-03-30

**Authors:** Isabelle A. M. van Thiel, Aimilia A. Stavrou, Auke de Jong, Bart Theelen, Mark Davids, Theodorus B. M. Hakvoort, Iris Admiraal-van den Berg, Isabelle C. M. Weert, Martine A. M. Hesselink-van de Kruijs, Duong Vu, Christine Moissl-Eichinger, Sigrid E. M. Heinsbroek, Daisy M. A. E. Jonkers, Ferry Hagen, Teun Boekhout, Wouter J. de Jonge, René M. van den Wijngaard

**Affiliations:** 1 Tytgat Institute for Liver and Intestinal Research, Amsterdam UMC location University of Amsterdam, Meibergdreef 9, Amsterdam, The Netherlands; 2 Amsterdam Gastroenterology Endocrinology Metabolism, Amsterdam, The Netherlands; 3 Westerdijk Fungal Biodiversity Institute, Utrecht, The Netherlands; 4 Institute of Biodiversity and Ecosystem Dynamics (IBED), University of Amsterdam, Amsterdam, The Netherlands; 5 Department of Medical Microbiology, University Medical Center Utrecht, Utrecht, The Netherlands; 6 Laboratory of Experimental Vascular Medicine, Amsterdam UMC location University of Amsterdam, Amsterdam, The Netherlands; 7 Microbiota Center Amsterdam, Amsterdam UMC location University of Amsterdam, Amsterdam, The Netherlands; 8 Division Gastroenterology-Hepatology, Department of Internal Medicine, NUTRIM School of Nutrition and Translational Research in Metabolism, Maastricht University Medical Center+, Maastricht, The Netherlands; 9 Diagnostic and Research Institute of Hygiene, Microbiology and Environmental Medicine, Center for Microbiome Research, Medical University Graz, Graz, Austria; 10 Gastroenterology and Hepatology, Amsterdam UMC location University of Amsterdam, Amsterdam, The Netherlands; 11 Department of General, Visceral, Thoracic and Vascular Surgery, University Hospital Bonn, Bonn, Germany

**Keywords:** Microbiology, Gastroenterology, Medical research

## Abstract

Irritable bowel syndrome (IBS) is a common disorder characterized by chronic abdominal pain and changes in bowel movements. Visceral hypersensitivity is thought to be responsible for pain complaints in a subset of patients. In an IBS-like animal model, visceral hypersensitivity was triggered by intestinal fungi, and lower mycobiota α-diversity in IBS patients was accompanied by a shift toward increased presence of *Candida albicans* and *Saccharomyces cerevisiae*. Yet, this shift was observed in hypersensitive as well as normosensitive patients and diversity did not differ between IBS subgroups. The latter suggests that, when a patient changes from hyper- to normosensitivity, the relevance of intestinal fungi is not necessarily reflected in compositional mycobiota changes. We now confirmed this notion by performing ITS1 sequencing on an existing longitudinal set of fecal samples. Since ITS1 methodology does not recognize variations within species, we next focused on heterogeneity within cultured healthy volunteer and IBS-derived *C. albicans* strains. We observed inter- and intra-individual genomic variation and partial clustering of strains from hypersensitive patients. Phenotyping showed differences related to growth, yeast-to-hyphae morphogenesis and gene expression, specifically of the gene encoding fungal toxin candidalysin. Our investigations emphasize the need for strain-specific cause-and-effect studies within the realm of IBS research.

## Introduction

IBS is the most prevalent disorder of gut-brain interaction affecting approximately 3–10% of the general population^1^. Abdominal pain is the hallmark symptom of this disorder. Because its pathophysiology is incompletely understood and likely has a multifactorial nature, development of effective treatment is challenging. In at least part of the IBS patients pain complaints are driven by visceral hypersensitivity, which is diagnosed as a decreased threshold of discomfort during colorectal balloon distensions^2,3^. Although gut microbes were suggested to contribute to visceral hypersensitivity, a possible role for intestinal fungi was largely ignored until recently^4,5^. Results obtained in the IBS-like maternal separation model of visceral hypersensitivity suggested causal involvement of the mycobiome. Earlier, the merits of this model became clear when findings on the role of mast cells and the histamine-1 receptor were successfully translated to IBS clinical trials^6–9^. Now, using fungicides, fecal transfers and targeting of yeast recognition, it was shown that mast cell dependent visceral hypersensitivity in the maternal separation model was mediated by gut yeast^10^. Furthermore, when comparing healthy volunteer and IBS fecal samples, decreased fungal diversity and an elevated relative abundance of *Candida albicans* and *Saccharomyces cerevisiae* were observed in IBS^10–13^. Yet, when comparing hypersensitive and normosensitive IBS patients, there were no differences in mycobiota α-diversity nor in the enhanced relative presence of *C. albicans* and *S. cerevisiae* between the two subgroups^10^. The latter may suggest that for yeast induced visceral hypersensitivity in IBS, genotypic and/or phenotypic dissimilarities on species level, are more relevant than quantitative alterations of dominant mycobiota species. *C. albicans* yeast strain variations within the human gut were recently described and it was shown that strain differences lead to distinct in vitro and in vivo immune responses^14–16^. Since immune (*i.e.* mast cell) activation was shown to be relevant in patients as well as in the yeast dependent visceral hypersensitivity model^6–10^, inter-strain differences may also influence visceral sensitivity status in IBS.

Although several groups now compared fecal mycobiota composition of healthy volunteers and IBS patients^10–13^, a comparison between hyper- and normosensitive patients was performed in one study only^10^. To further address the reported absence of overt IBS sub-type differences in mycobiota composition and to accentuate the possible relevance of *C. albicans* inter-strain diversity, we decided on a two-fold approach. First, we assessed the IBS gut micro- and mycobiota in a set of longitudinal fecal samples by comparing patients that changed from visceral hypersensitivity to normosensitivity, with those that remained hypersensitive in time. Second, we used an additional cohort to assess possible yeast strain variation, by genotyping and functional assays, in cross-sectional fecal isolates of *C. albicans*. Our results indicated that (i) a change in visceral sensitivity status is not necessarily manifested by alterations in intestinal mycobiota composition, and (ii) genetic and phenotypic diversity of fecal *C. albicans* strains occurs within and between patients. Thus, we propose that investigations regarding the importance of the gut mycobiome, should not be limited to composition, but additionally focus on strain-level variability.

## Results

### Patients are stratified based on outcome rather than treatment allocation

Fecal samples used for myco- and microbiota analysis were gathered from an already completed clinical study. In total we used pre- and post-therapy samples of 16 IBS patients, all of which were hypersensitive to rectal distension at start^17^. This is a small but unique sample set, mainly because patients were subjected to 2 rectal barostats in a 6 week time interval. The original study design by Ludidi et al*.* consisted of a double-blinded intervention with multispecies probiotics aiming to improve visceral hypersensitivity. No significant effect of a 6 weeks probiotic intervention over placebo was observed with regard to primary outcome. Here, prior to proceeding on outcome-based sample stratification, we investigated whether treatment affected ITS1-based gut mycobiota profiles. Analysis of samples indicated that Shannon Diversity was generally higher in patients allocated to probiotics (Supplementary Fig. 1a,b. Linear mixed effect models (LME); *Treatment*, F = 13.6, *p* = 0.006), indicating incomplete randomization and introduction of bias for further analysis. The difference in Shannon diversity persisted after 6 weeks and associated with altering sensitivity status (Supplementary Fig. 1a,b. LME; *Treatment : Time Point : Outcome* F = 8.0, *p* = 0.020). However, this observation is influenced by the aforementioned bias. Moreover, treatment had no influence on mycobiota composition as expressed in multilevel principle component analysis (mPCA, Supplementary Fig. 1c). Knowing that multispecies probiotic treatment was also without significant effect on visceral hypersensitivity^17^, we decided to group patient samples based on change of visceral sensitivity status (improvers *vs.* non-improvers) irrespective of initial treatment allocation. Groups were similar by means of allocated treatment, age, IBS subtype, body mass index, bowel movements per day and symptoms scores (Table 1).

**Table 1:** Patient characteristics for microbial composition analysis.

General characteristics		Non-improvers	Improvers
		*n* = 8	*n* = 8
Age	mean ± SD years	47.0 ± 9.2	44.9 ± 14.3
Sex	*n* (% females)	6 (75.0%)	5 (62.5%)
Body Mass Index	mean ± SD kg m^-2^	23.9 ± 3.7	24.0 ± 2.1
Treatment	*n* (% probiotic)	4 (50.0%)	3 (37.5%)
IBS subtype	IBS-C	1 (12.5%)	1 (12.5%)
	IBS-D	5 (62.5%)	4 (50.0%)
	IBS-M	2 (25.0%)	2 (25.0%)
	IBS-U	–	1 (12.5%)
**Symptoms and sensitivity at baseline**	mean ± SD		
Mean composite symptom score^a^		14.5 ± 4.1	15.2 ± 3.7
Nausea		2.1 ± 1.2	2.1 ± 1.2
Cramping		2.5 ± 1.1	2.9 ± 1.2
Abdominal pain		2.8 ± 0.8	2.5 ± 1.3
Bloating		3.3 ± 1.2	3.0 ± 1.2
Flatulence		2.5 ± 1.0	2.7 ± 0.7
Stools with mucus		1.2 ± 0.4	1.4 ± 0.9
Stools with blood		1.0 ± 0.0	1.1 ± 0.2
Max VAS score for discomfort 0–23 mmHg distension^b^		55.0 ± 27.9	49.9 ± 32.0
Max VAS score for pain 0–23 mmHg distension^b^		51.3 ± 35.7	42.0 ± 28.9
**Symptoms and sensitivity at 6 weeks**	mean ± SD		
Mean composite symptom score ^a^		14.8 ± 4.5	11.3 ± 3.8
Nausea		2.2 ± 1.3	1.5 ± 0.6
Cramping		2.6 ± 1.2	2.0 ± 0.9
Abdominal pain		2.7 ± 1.2	2.1 ± 0.9
Bloating		2.9 ± 1.2	2.6 ± 1.0
Flatulence		2.4 ± 1.1	2.1 ± 0.3
Stools with mucus		1.0 ± 0.0	1.0 ± 0.0
Stools with blood		1.0 ± 0.0	1.0 ± 0.1
Max VAS score for discomfort 0–23 mmHg distension^b^		57.5 ± 30.5	29.9 ± 19.2
Max VAS score for pain 0–23 mmHg distension^b^		54.8 ± 30.6	6.0 ± 3.1

Patient samples were selected from a previous clinical trial^17^. At baseline, all patients were diagnosed as hypersensitive to colorectal distension. Patients are separated based on improvers vs. non-improvers, referring to a change of sensitivity status to normosensitive or remaining hypersensitive.

*BMI* body mass index, *BM* bowel movements. *IBS subtypes U, C, D, M* unspecified, constipation, diarrhea, mixed defecation pattern.

^a^composite score calculated based on mean of 7 subjects scored on a 5-point Likert scale from 1 to 5 (i.e. nausea, cramping, abdominal pain, bloating, flatulence, stools with mucus, stools with blood) for 14 days. Composite score range 7–35.

^b^Maximum VAS for pain 0–23 mmHg ≥ 10 mm is considered as hypersensitive to colorectal distension.

### No association of the mycobiota with improvement of sensitivity in IBS

**Figure 1:**
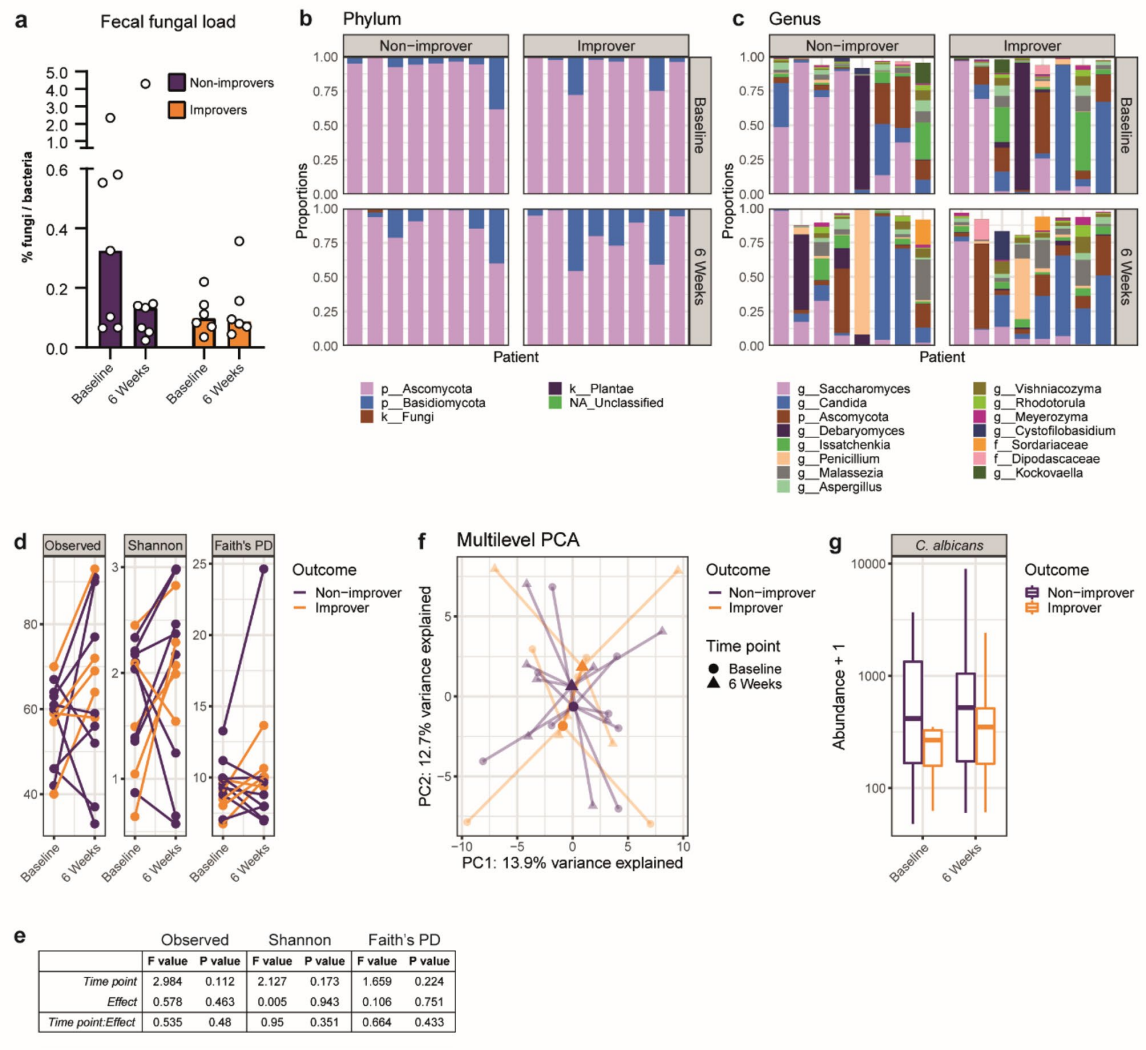
Fecal mycobiota analysis reveals no differences with respect to sensitivity status. (**a**) Fungal load determination by FungiQuant qPCR. (**b**), (**c**) Taxonomic analysis of sequenced samples on relative abundance of top 5 phyla (**b**) and top 15 genera (**c**). (**d**), (**e**) α-diversity metrics according to number of observed species, Shannon diversity, Faith’s Phylogenetic Diversity (PD). Linear Mixed Effects model results presented in (**e**). (**f**) Community analysis by multilevel principal component analysis. Bold symbols represent centroids of each sample group, while light symbols indicate individual samples. (**g**) Abundance of *Candida albicans* based on ITS1 sequencing reads.

Altered mycobiota compositions were previously described for patients with IBS, but fungal load was never quantified. We determined abundances of fungal and bacterial DNA using the qPCR-based FungiQuant approach^18^. At baseline and at the six weeks’ time point, fungal load of improvers did not significantly differ from non-improvers (Fig. 1a). Subsequent taxonomic analysis of the ITS1 amplicons indicated that the vast majority of reads is allocated to the phylum *Ascomycota* throughout all samples (Fig. 1b). Analysis of the top 15 genera showed diverse mycobiota profiles, with the most abundant genera being *Saccharomyces* spp. and *Candida* spp. (Fig. 1c). No significant differences in α-diversity metrics were observed between patient groups or time points (Fig. 1d,e). In addition, no differences in fecal mycobiota composition were found when comparing improvers and non-improvers (Fig. 1f). Although *C. albicans* was the most abundant species among *Candida* spp. (Supplementary Fig. 2), its abundance was similar between improvers and non-improvers (Fig. 1g).

Taken together, based on abundance and composition of the gut mycobiota at taxonomic levels down to and including species level, no associations were observed with changing visceral sensitivity status. These data are in line with our earlier investigations, where we observed increased abundances of *C. albicans* and *S. cerevisiae* in patients with IBS compared to healthy individuals, but not when comparing hyper- and normosensitive patient subsets^10^.

### Fecal *C. albicans* strains show both inter- and intra-individual genomic variations.

ITS1-based mycobiota analysis provides a comprehensive view of fungal communities, but its relevance is also limited due to the assignment of sequencing reads to amplicon sequence variants (ASVs). Strain variants, *i.e.* strain sub-species, cannot usually be recognized by ITS1 sequencing. Yet, *C. albicans* strain variations can lead to distinct immune responses, and previous reports indicated that strain variants can be identified between and even within individuals^14–16^. Therefore, even though we observed no apparent differences in fungal composition or abundance, we were interested to inventory *C. albicans* sub-species in IBS and healthy volunteer fecal samples.

**Figure 2:**
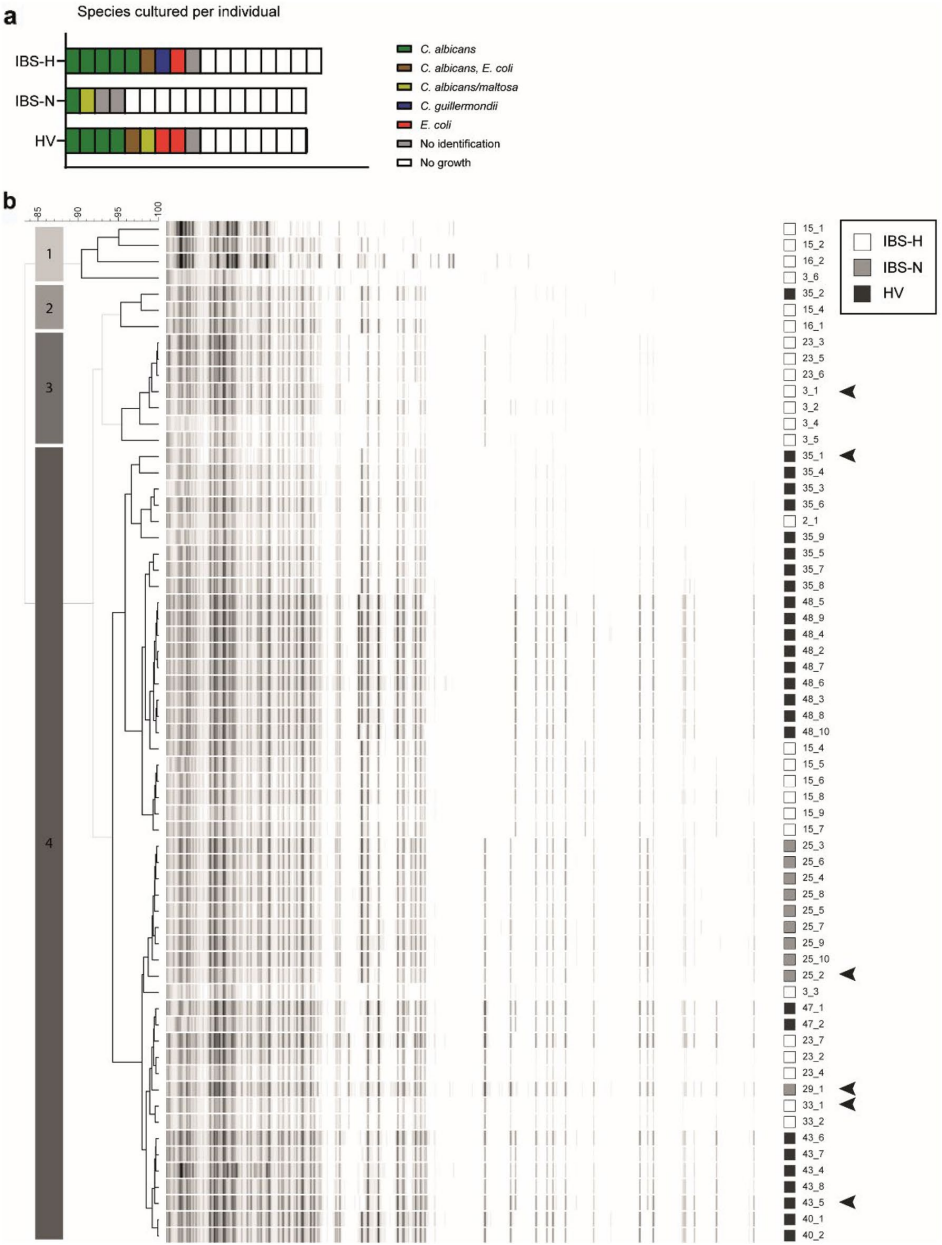
Culture of fecal samples yields genetically diverse *C. albicans* strains. (**a**) Cultured species from fecal samples as determined by MALDI-TOF MS analysis. Each rectangle indicates one patient sample. (**b**) Amplified Fragment Length Polymorphisms (AFLP) fingerprint analysis of 63 *C. albicans* strains reveals genetic differences as reflected in varying fingerprint patterns. Colored squares indicate sample group origin of the strain, composed numbers identify fecal sample and strain number respectively. Dendrogram shows clustering of strains into 4 groups at 90% similarity. Arrowheads indicate strains selected for follow-up phenotypic examination. *IBS-H* hypersensitive IBS patients, *IBS-N* normosensitive IBS patients, *HV* healthy volunteer.

The need for inclusion of healthy volunteer samples led us to use a second, independent sample set, containing both hypersensitive (IBS-H) and normosensitive (IBS-N) patients, as well as a group of healthy volunteers (HV)^10^. Based on general characteristics, groups were of similar nature (Table 2). Fecal samples were gathered from −80 ℃ and cultured aerobically on solid culture media. Prolonged frozen storage likely influenced microbial viability, but still nearly half of all samples showed growth of at least one microbial species (45%, Fig. 2a). Depending on the number of colonies per plate, a maximum of ten morphologically similar colonies per patient were identified by MALDI-TOF MS. The most frequently identified fungal species was *C. albicans* (64% of all positive cultures, Fig. 2a), which was in line with previous findings^13^.

**Table 2:** Patient characteristics for fungal genetic and phenotype analysis.

General characteristics		IBS-H	IBS-N	HV
		*n* = 17	*n* = 16	*n* = 16
Age	mean ± SD years	39.8 ± 19.7	52.6 ± 18.3	44.7 ± 15.5
Sex	*n* (% females)	13 (76.5%)	12 (75.0%)	8 (50.0%)
Body Mass Index	mean ± SD kg m^-2^	24.0 ± 4.7	25.0 ± 4.0	24.4 ± 5.1
IBS subtype	IBS-C	5 (29.4%)	4 (25.0%)	–
	IBS-D	4 (23.5%)	7 (43.8%)	–
	IBS-M	8 (47.1%)	2 (25.0%)	–
	IBS-U	0 (0.0%)	1 (6.3%)	–
**Symptoms and sensitivity**	mean ± SD			
Mean composite symptom score^a^		20.5 ± 5.2	16.3 ± 4.1	9.9 ± 1.2
Discomfort		2.9 ± 0.8	2.2 ± 0.7	1.1 ± 0.2
Pain		2.7 ± 0.8	1.9 ± 0.6	1.1 ± 0.1
Nausea		2.3 ± 1.2	1.6 ± 0.6	1.10 ± 0.0
Bloating		2.4 ± 0.7	2.0 ± 0.9	1.1 ± 0.2
Flatulence		2.3 ± 1.0	2.3 ± 1.0	1.3 ± 0.4
Belching		1.8 ± 0.8	1.4 ± 0.6	1.1 ± 0.3
Constipation		1.7 ± 0.7	1.3 ± 0.5	1.1 ± 0.1
Diarrhea		1.6 ± 0.7	1.3 ± 0.3	1.0 ± 0.1
Overall symptom burden		2.8 ± 0.8	2.3 ± 0.6	1.1 ± 0.2
Max VAS score for discomfort 0–26 mmHg distension^b^	mean ± SD	21.1 ± 20.7	38.2 ± 30.8	17.7 ± 17.3
Max VAS score for pain 0–26 mmHg distension^b^	mean ± SD	50.7 ± 23.3	5.3 ± 5.9	1.3 ± 2.3

Patients recruited from Maastricht IBS (MIBS) study cohort.

^a^Composite score calculated based on 14-day mean of 9 subjects scored on a 5-point Likert scale. Each subject was scored from 1 to 5 (i.e. abdominal discomfort, abdominal pain, nausea, bloating, flatulence, belching, constipation, diarrhea, symptom burden). Composite score range 9–45.

^b^Maximum VAS for pain 0–26 mmHg ≥ 20 mm is considered as hypersensitive to colorectal distension.

To assess genetic variation, Amplified Fragment Length Polymorphisms (AFLP) fingerprint analysis was performed on 63 *C. albicans* strains that were cultured from *n* = 6 IBS-H, *n* = 2 IBS-N and *n* = 4 HV fecal samples. As expected, AFLP fingerprinting showed inter-individual genetic strain diversity (Fig. 2b). Based on a 90% similarity cut-off we observed 4 separate clusters (left column) and, with the exception of HV strain 35_2, all strains in clusters 1, 2 and 3 were cultured from IBS-H samples. The latter may suggest strain-specific causality in regards to visceral hypersensitivity. Prominent intra-individual diversity, *i.e.* strains derived from a single fecal sample but distributed over different AFLP-clusters, was observed in 4 IBS-H patients (numbers 3, 15, 16 and 23) and 1 HV (number 35). Due to low numbers however, suggestions on possible association between high intra-individual diversity and visceral hypersensitivity, remain speculative.

### Isolated *C. albicans* strains possess different phenotypic characteristics

**Figure 3:**
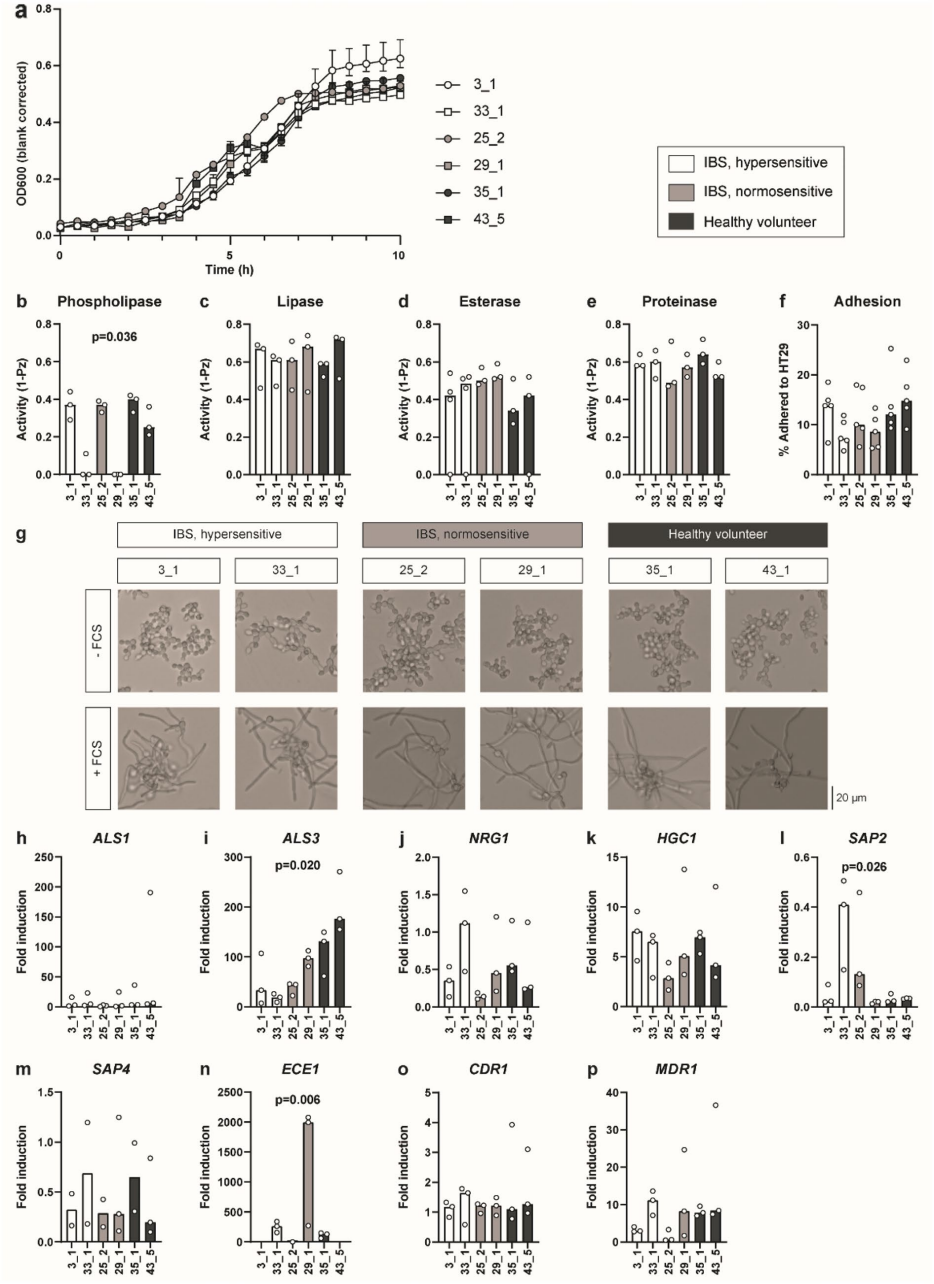
Phenotypic assessment of fecal *C. albicans* isolates reveals strain-level differences. (**a**) Growth curve of 6 selected *C. albicans* strains. IBS-H, hypersensitive IBS patients; IBS-N, normosensitive IBS patients; HV, healthy volunteer. Data shown as median and range (*n* = 3). (**b**)–(**e**) Phenotypic assessment of phospholipase, lipase, esterase, and protease activity. Activity is determined by measuring halo size relative to colony size (*n* = 3–4). (**f**) Adhesion of yeast strains to HT-29 colon carcinoma monolayers, expressed as percentage of added cells (*n* = 5). g) Representative photographs of FCS-induced morphogenesis. Magnification 20 × . (**h**)–(**p**) Induction of hyphae-formation related genes, virulence factors, and drug-resistance genes upon addition of FCS, expressed as fold induction relative to unstimulated yeast samples. Experiment performed in triplicate, missing values are due to no detection of transcript. Significances tested using Kruskal–Wallis test. Data shown as median and individual datapoints. FCS, fetal calf serum.

To address phenotypical diversity, we selected 2 strains in each group (IBS-N, IBS-H and HV), based on maximum within-group distances of AFLP fingerprints. The selected strains are indicated by arrowheads in the right side column of Fig. 2b. When registering ten hour growth profiles, we observed dissimilar proliferation characteristics (Fig. 3a). Strains 29_1 (IBS-N), 33_1 (IBS-H), and 43_5 (HV) showed a remarkable plateau halfway through the exponential growth phase. This may be due to a diauxic shift, i.e. switching of primary metabolic source, as is frequently observed in yeast^19^. We next assessed various traits associated with virulence, including secretion of virulence-associated enzymes and morphogenesis^20^. Upon evaluation of secreted enzymes under standard culture conditions, only phospholipase activity was significantly different (Fig. 3b; *p* = 0.036). Secretion of lipases, esterases and proteinases was similar throughout the six strains (Fig. 3c–e). Next, we quantified yeast adhesion to HT-29 colon carcinoma-derived epithelial cells. Although strains 3_1 (IBS-H), 35_1 and 43_5 (both HV) appear to have higher adhesive capacity, results obtained in a total of 5 independent experiments showed no significant differences (Fig. 3f; *p* = 0.09).

Transcriptomic profiles of invasiveness markers are known to change during biofilm formation^14^. Since intestinal *C. albicans* occurs both in yeast and hyphal states^21^, we assessed possible differences in serum-induced yeast-to-hyphae morphogenesis. Four hours after serum addition, hyphae induction rate and appearance were similar in all 6 strains (Fig. 3g). Upregulation of genes associated with adhesion to cells (*ALS1*, *ALS3*)^22^, regulation of hyphae formation (*HGC1*, *NRG1*)^23^, aspartic peptidases (*SAP2*, *SAP4*)^24^, the gene encoding the fungal toxin candidalysin (*ECE1*)^25^ and drug-resistance related genes (*MDR1*, *CDR1*)^26^ were assessed through qPCR. Normalized expression (*i.e.* compared to FCS-unstimulated yeast) was significantly different for *ALS3*, *SAP2*, and *ECE1* (Fig. 3h–p; *p* = 0.020, *p* = 0.026, *p* = 0.006 respectively). Remarkably, while *ECE1* expression showed high induction rates in strains 33_1 (IBS-H), 29_1 (IBS-N) and 35_1 (HV), expression levels were nearing lower detection limit in the remaining 3 strains. Our combined in vitro data indicate that, in addition to genotypic diversity, intestinal yeast strains display distinctive phenotypical characteristics. Both are inevitably overlooked by ITS1 sequencing approaches. Yet, depending on the local microenvironment, they may be relevant to IBS endotype differences like presence or absence of visceral hypersensitivity.

### Bacterial diversity and composition are associated with normalization of visceral sensitivity status

**Figure 4:**
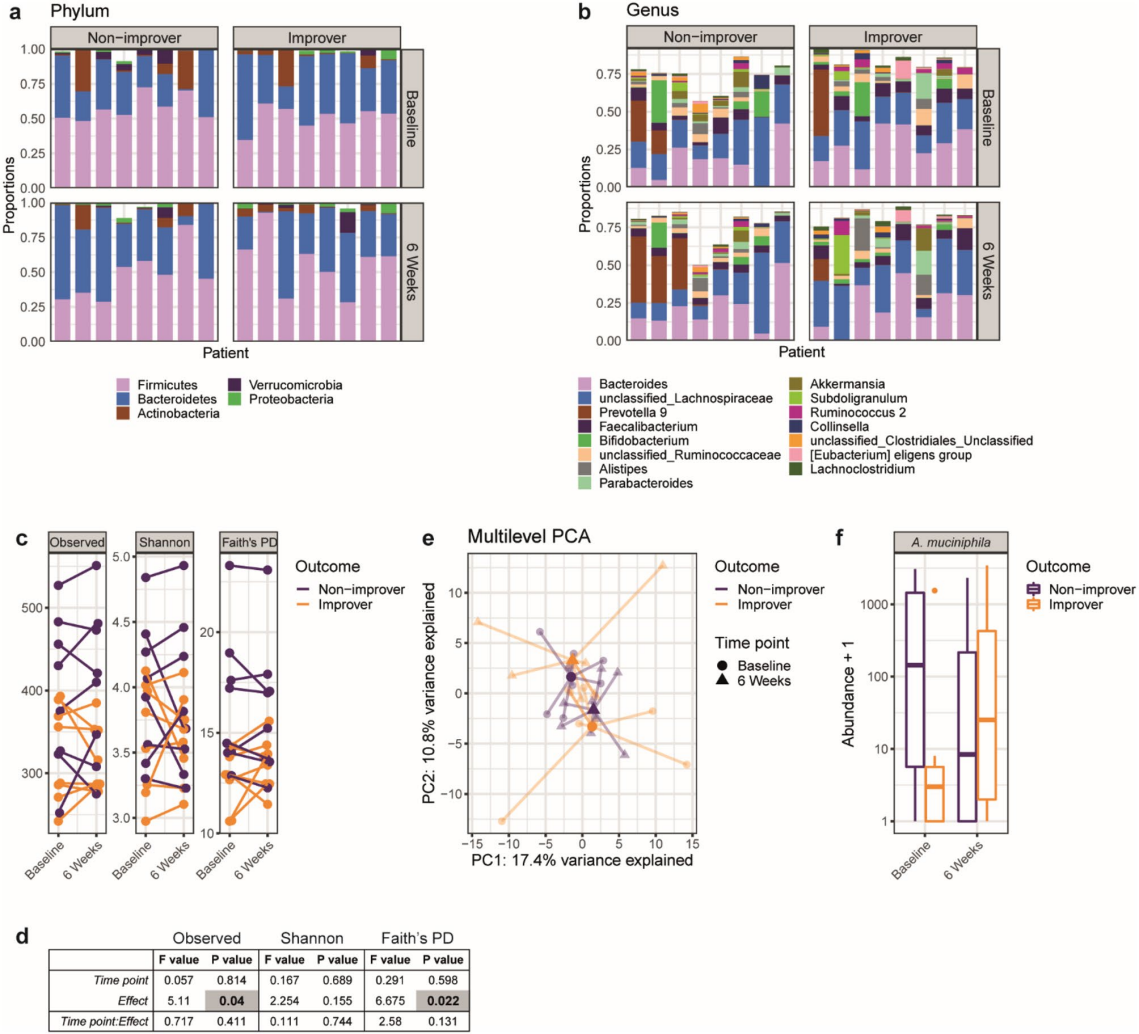
Fecal bacterial microbiome analysis indicates association of α-diversity metrics, composition, and *Akkermansia muciniphila* with changing sensitivity status. (**a**), (**b**) Taxonomic representation of all samples on relative abundance of top 5 phyla (**a**) or top 15 genera (**b**). Patients are grouped based on a normalization of sensitivity status (improvers), or unaltered sensitivity status (non-improvers) over the course of six weeks. Each column represents paired samples of one patient at baseline and 6 weeks. (**c**), (**d**) Microbial richness described according to α-diversity metrics Observed species, Shannon index, and Faith’s Phylogenetic Diversity (PD). Observed species and Faith’s PD are generally lower for improvers. Results of linear mixed effects model presented in (**d**). (**e**) Community analysis by multilevel principle component analysis (*p* = 0.002, PERMANOVA). Bold symbols represent centroids of each sample group, while light symbols indicate individual samples. (**f**) Abundance of *Akkermansia muciniphila* is associated with the change in sensitivity status (*p* = 0.007, LME).

In comparison to the gut mycobiota, the bacterial microbiota has received massive attention in past studies. Compared to healthy volunteers, bacterial community composition in IBS is frequently reflected by lower diversity metrics^4,27^. Although small, our sample set is rather unique because it couples longitudinal sampling to barostat measurements. Hence, we additionally performed 16S rRNA gene-based microbiota analysis on all available samples of the probiotics trial (Table 1)^17^. Taxonomic evaluation of the top five phyla revealed high relative abundance of Firmicutes and Bacteroidetes consistently throughout all sample pairs (Fig. 4a). On genus level *Bacteroides*, *Lachnospiracae*, and *Prevotella* were most abundant within the top 15 genera (Fig. 4b). Probiotics treatment altered Shannon diversity (Supplementary Fig. 3a,b; LME, *Time point : Treatment*, F = 6.0, *p* = 0.031), and induced minor compositional change (Supplementary Fig. 3c; PERMANOVA, F = 3.2, *p* = 0.007). However, as stated before, these treatment-induced changes were not associated with clinical outcome^17^.

With respect to changing sensitivity status, improvers showed lower α-diversity metrics (Fig. 4c,d; Observed species F = 5.11, *p* = 0.04; Faith’s PD F = 6.675, *p* = 0.022). In addition, microbiome composition changed significantly over time (Fig. 4e; PERMANOVA, F = 3.2, *p* = 0.002). We sought for differentially abundant bacterial taxa that could serve as a marker for changing sensitivity status. Unbiased analysis did not yield any hits due to trespassing the false discovery rate cut-off. Based on the mPCA loading plot however (Supplementary Fig. 4), the phylum *Verrucomicrobia* showed a distinct location on both principal components (PC) PC1 and PC2. A member of this phylum, *Akkermansia muciniphila*, is a well-known mucus-degrading bacterium, frequently described in association with overall health (*e.g.* leanness, metabolism, and decreased inflammatory processes)^28^. Here, abundance of *A. muciniphila* associated with change of sensitivity status (Fig. 4f; LME, *p* = 0.007). Initial low abundance followed by enhanced abundance of *A. muciniphila* appears to be favorable for change to normosensitivity. This observations seems to confirm earlier results on the inverse correlation between relative abundance of *A. muciniphila* and pain reduction that was described upon fecal microbiota transfer in an open-labeled IBS study^29^.

### Archaeal abundance was not associated with sensitivity status

In addition to bacteria and fungi, low-abundant gas-forming archaea were suggested to play a role in IBS complaints^30,31^. In our clinical cohort, sequencing of archaeal 16S rRNA gene resulted in a highly variable number of reads (Supplementary Fig. 5). Hence, no archaeal signature associated with visceral sensitivity could be discriminated in these samples.

## Discussion

In longitudinal fecal samples obtained from patients with IBS and altering visceral sensitivity status, we were unable to link ITS1-based gut mycobiota composition to changing sensitivity. For detailed analysis on sub-species level, we then performed AFLP fingerprinting on a large set of *C. albicans* strains that were cultured from healthy volunteer and hyper- and normosensitive IBS fecal samples. Our results showed inter and intra-individual genetic variation and separate clustering of strains derived from hypersensitive IBS patients. Subsequent characterization of a genetically diverse sub-selection of *C. albicans* strains indicated differences in growth characteristics and differential expression of virulence-associated enzymes and genes. Such genetic and phenotypic diversity at strain level remains unnoticed when using ITS1 sequencing approaches. Whether the observed in vitro differences are relevant for visceral pain remains to be established. Our results do however emphasize that a focus on gut mycobiome composition alone can never rule out possible contribution of intestinal yeast to visceral hypersensitivity in IBS.

Visceral hypersensitivity is broadly investigated as a key determinant of abdominal pain in IBS^2^. Measuring visceral sensitivity, or changes thereof, by means of rectal barostat is both invasive and laborious. This may explain why clinical trials involving this methodology are often of small sample size, and why mycobiota profiles of IBS patients with changing sensitivity status were never characterized before. Yet, we obtained a small set of longitudinal fecal samples, gathered from hypersensitive IBS patients that either improved their sensitivity status or remained hypersensitive in time^17^. Our analysis of ITS1 sequencing results showed that a change from hyper- to normosensitivity was not accompanied by altered mycobiome composition. This result was in line with our previous investigations where, despite differences between healthy volunteers and IBS patients, mycobiota α-diversity did not differ when comparing hypersensitive with normosensitive patients^10^. Similarly, while Das et al*.* described dissimilar mycobiota beta-diversity when comparing healthy volunteers and IBS patients, they observed no difference between clinical subtypes of IBS^11^. These findings may suggest a lack of importance for the gut mycobiome. However, in an IBS-like animal model with proven translational value^6–9^ we observed a crucial role for intestinal fungi in development and maintenance of visceral hypersensitivity^10,32,33^. Furthermore, while within-species strain level diversity may be highly relevant, ITS1 sequencing approaches used in these mycobiota composition studies cannot reveal the presence of different strains. We concluded that, in addition to bulk assessment of fungal DNA, the role of intestinal fungi should also be addressed at strain level.

We cultured feces derived yeasts form hyper- and normosensitive IBS patients as well as healthy volunteers and decided to focus on strain diversity within the *C. albicans* species. Strain differences of *C. albicans* were previously found to be relevant in other (intestinal) diseases and host-microbe interactions^14–16^. Moreover, elevated abundance of the genus *Candida* (spp. and *C. albicans)* was found in patients with IBS^10,11^ and correlated with bloating in patients with diarrhea-predominant IBS^12^. To assess the degree of genetic variability in our *C. albicans* culture collection we performed AFLP fingerprinting and, as expected, observed inter- and intra-individual strain diversity. The AFLP phenogram divided a total of 63 strains into 4 different clusters. Importantly, 3 of these clusters were almost exclusively occupied by *C. albicans* strains cultured from patients with established visceral hypersensitivity. Clustering of these strains may suggest strain-specific causality in regards to visceral hypersensitivity, but could also be the result of *C. albicans* genotype selection due to unknown IBS-H-specific host influences. In a recent study, Sciavilla et al*.* performed random amplification of polymorphic DNA (RAPD)-fingerprinting on IBS and healthy volunteer derived *C. albicans* strains^13^. Although this study also suggested distinct clonal expansion in IBS, it is not possible to extrapolate results to visceral hypersensitivity because this patient cohort was not subjected to rectal barostat. Moreover, only 35% of their patients was diagnosed with abdominal pain complaints, and these individuals cannot be singled out from the results section. Nevertheless, the combined results of the two studies emphasize the relevance of strain specific investigations regarding the role of yeast in IBS.

AFLP and RAPD fingerprinting are powerful techniques, but they do not provide any clues on possible phenotypic consequences of the observed differences. We decided to phenotype a subset of genetically distinct strains from our clinical *C. albicans* collection. The reaction of the host towards a fungal pathogen relies on multiple factors, and virulence factors play an important role. Even when strains do not differ significantly in vitro, they may still possess differential virulence in vivo^16^, in part explained by expression levels of virulence proteins^21^. In our strains, we observed differential expression of *ALS3*, *SAP2*, and *ECE1* which are involved in adhesion and the generation of a cytolytic fungal toxin called candidalysin, respectively^22,25^. In this small exploratory study, we cannot be sure which characteristics are important or not, but *ECE1* was previously shown to be crucial for epithelial damage^34^. In IBS, candidalysin may induce epithelial barrier dysfunction and subsequent mast cell activation that were both shown to be relevant in this disorder^6,9,35^. Evidently, a higher number of strains would be needed to establish associations between yeast phenotype and visceral sensitivity status.

Genetic differences of the *C. albicans* strains may also be expressed in the gut microenvironment through mechanisms beyond virulence. Hong et al*.* showed a positive correlation between *Candida* and severity of bloating in patients with IBS^12^. In our hands, the growth curve of half of the evaluated *C. albicans* strains showed a possible diauxic shift, defined as a switch from main energy source as frequently researched in *S. cerevisiae*^19^. Prior to such a switch, and under aerobic conditions, the main energy carbon-based source glucose is fermented into ethanol. Then, when culture medium becomes deprived of glucose, yeast cells switch to respiratory consumption of ethanol, explaining the lag phase in proliferation. In our experiments we did not monitor ethanol production. But, fermentation of glucose in the presence of oxygen, the so called Crabtree effect, has been described for *C. albicans* as well^36^. Regardless, the colonic lumen holds an anaerobic environment, and the relevance for IBS of these aerobically established strain differences may be questioned. Even so, our data showed different kinetics in primary carbon source utilization. Future investigations should address whether such differences also apply to fermentation in the colon. Limitation of fermentable sugars (low FODMAP, oligosaccharides, disaccharides, monosaccharides, and polyols) is a common dietary intervention for patients with IBS to reduce bloating through microbial fermentation of carbohydrates^37^. Strain variations in fermenting capacity may add to explain why the low FODMAP diet is successful in part of the patient population only.

In this study we examined the longitudinal gut mycobiota in relation to changing visceral sensitivity status of patients with IBS. Amongst others, our results showed the absence of overt alterations in mycobiome composition when patients changed from hyper- to normosensitivity. Yet, this ‘negative-finding’ may relate to low sample numbers used. This is an important limitation of the study that relates to the invasive and lengthy procedure needed to assess visceral (hyper)-sensitivity. As a result, large cohorts of patients, subjected to multiple barostat measurements, are not available. In order to increase numbers, future investigations may assess abdominal pain scores by questionnaires instead. A similar, numbers related, caveat exists for our pioneering investigations on *C. albicans* strain differences. Using AFLP fingerprinting, we showed genetic variation among strains and partial clustering of strains isolated from hypersensitive patients. For subsequent phenotyping we then selected a subset of only six strains to reflect three investigational sample-groups. Clearly, this low number prevented the meaningful assignment of strain differences to health status. Our choice was motivated by the number of phenotypical assays that were performed in order to identify promising targets for future investigations. Already in this limited set of strains, we were able to show different expression of virulence factors like candidalysin and, possibly, dissimilar substrate usage during proliferation. Future efforts to address further these particular findings should include higher numbers of *C. albicans* isolates. Concerning candidalysin, we expect that experiments similar to those performed by Allert et al*.* will provide evidence that differential expression of this cytolytic toxin leads to differences in gut barrier function and fungal translocation^34^. In relation to substrate usage, it should be re-emphasized that we conducted our proliferation experiments in aerobic conditions. Future investigations should apply anaerobic conditions and focus on fermentation instead of proliferation. The former may show that, even when provided with the same substrate, fermentation kinetics and related gas production may differ among strains. A further increase of potentially interesting investigational targets may derive from whole genome sequencing of *C. albicans* strains. In contrast to AFLP, such an approach could facilitate identification of polymorphisms that correlate with health or disease, which may open up even newer avenues of phenotypical investigations.

Despite the aforementioned limitations, we showed that mycobiota composition may not alter with changing sensitivity status, and herewith confirmed that mycobiota analysis cannot separate hypersensitive from normosensitive patients. Nonetheless, we observed strain differences of *C. albicans* obtained from healthy volunteers and normo-, and hypersensitive patients with IBS. Most prominently, we observed partial clustering of IBS-H derived and AFLP fingerprinted *C. albicans* strains. Although we also observed phenotypic diversity, due to the low number of cultures used in the present study, it is not possible to correlate strain diversity with disease phenotype. Yet, our exploratory investigations on yeast genetic make-up, virulence factors and growth characteristics provide basis for further in vitro and pre-clinical investigations of the gut mycobiome in IBS on a sub-species level.

## Materials and methods

### Patients and fecal samples

Frozen fecal samples, collected during a controlled probiotic intervention study of Maastricht University Medical Center + , the Netherlands, were selected for genomic analysis^17^. Patients hypersensitive to colorectal distension were included in a multispecies probiotic study with improvement of visceral perception as primary endpoint. Sensitivity to colorectal distension was determined at baseline and after six weeks of intervention. Barostat measurements were described by Ludidi et al.^17^ and visceral hypersensitivity was defined as a Visual Analogue Scale (VAS) score > 10 mm at pressure step 23 mmHg^38^. Symptom severity was scored for seven symptoms (i.e. flatulence, abdominal pain, abdominal cramping, nausea, bloating, stools with blood, stools with mucus) on a 5-point Likert scale for 14 days. Multispecies probiotic treatment was not superior over placebo^17^. Consequently, in the present study, groups were regarded based on the outcome of the study: eight patients of whom sensitivity status normalized were selected (*n* = 3 probiotic, *n* = 5 placebo). A second group of eight patients was composed based on persistent visceral hypersensitivity and treatment (*n* = 4 placebo; *n* = 4 probiotic). Groups were not matched otherwise (e.g. age, gender, symptom severity).

The second collection of samples, employed for fungal cultures and genotyping, were retrieved from the Maastricht IBS (MIBS) cohort study. Fecal samples were collected at home, kept cool, delivered within 48 h from production, and stored at − 80 ℃ until further usage. Symptom severity was scored for 14 consecutive days on nine symptoms (i.e. abdominal pain, abdominal discomfort, bloating, belching, nausea, flatulence, constipation, diarrhea, and overall symptom burden) using a 5-point Likert scale. Visceral hypersensitivity was defined as a VAS score ≥ 20 mm at pressure step 26 mmHg^39^.

All included subjects gave written informed consent. Procedures were approved by the Maastricht University Medical Center + Committee of Ethics and performed according to the revised Declaration of Helsinki (59th General Assembly of the WMA, Seoul, South Korea, October 2008). Both studies have been registered in the US National Library of Medicine (http://clinicaltrials.gov). The probiotics study (NCT00702026) was registered on June 19, 2008 and the Maastricht IBS cohort (NCT00775060) on October 17, 2008.

### Microbial DNA isolation

Microbial DNA from feces was isolated using the PSP Spin Stool DNA Plus Kit (Invitek Molecular/Isogen) as previously described^40^. Samples were obtained by scraping − 80 ℃ frozen stool, which were subsequently homogenized (MilliPore Lysing Matrix E, Bio-Connect) in Stool DNA Stabilizer buffer using a FastPrep bead beater (3 × 30 s, 6.5 ms^-2^. MP Biomedicals, Abcoude, The Netherlands). Samples were heated at 95 ℃ for 15 min followed by cooling on ice. Supernatants were transferred to the PSP InviAdsorb tubes and manufacturer’s protocol was followed from this step on. Negative procedural controls were treated in the same way. Total DNA concentrations were determined using Qubit fluorometric Quantitation method (ThermoFisher Scientific, Landsmeer, The Netherlands).

### Bacteria-specific 16S rRNA gene sequencing and data processing

Determination of bacterial composition was performed as previously described^41,42^. A one-step procedure targeting the V3-V4 region of the 16S rRNA gene was executed by Microbiota Center Amsterdam (MiCA). Libraries were sequenced on a MiSeq platform (Illumina, Eindhoven, The Netherlands) in a paired-end fashion for 250 cycles using V3 chemistry. Resulting reads were trimmed and merged using USEARCH^43^. Next, reads were passed through quality control, in which reads were discarded if they did not pass the Illumina chastity filter, had expected errors (i.e. error rate > 2), or if reads were shorter than 380 bases. Amplified Sequence Variants (ASVs) were called if a minimum abundance of 4 reads per ASV was observed within a sample^43^. Reads were mapped against the collective ASV database to determine abundances. IDTaxa^44^ and SILVA 16S ribosomal database V132^45^ were used to assign taxonomy.

### Fungal-specific ITS1 sequencing and data processing

Fungal composition was determined by internal transcribed spacer 1 (ITS1) amplicon sequence analysis as previously described^42^. In summary, fecal DNA was amplified by a two-step PCR to produce amplicon libraries. Phusion High Fidelity DNA polymerase and ITS1 primers with overhang for the Illumina Nextera platform were used in duplicate: [sense] 5’-TCGTCGGCAGCGTCAGATGTGTATAAGAGACAGCTTGGTCATTTAGAGGAAGTAA and [antisense] 5’- GTCTCGTGGGCTCGGAGATGTGTATAAGAGACAGGCTGCGTTCTTCATCGATGC. Pooled duplicates were purified using Agencourt AMPure XP Bead system and resuspended in DNA-free water. In the second amplification step, multiplex indices and Illumina sequencing adapters were introduced using the Kapa polymerase system. The resulting amplicons (200–700 bp) were purified twice using the AMPure system and taken into DNA-free water. Concentrations were determined using the Qubit fluorometric quantitation method and quality of the samples was determined using the Agilent Bioanalyzer DNA-1000 chip (Amstelveen, the Netherlands). DNA concentration was used to mix the indices (i5 and i7) tagged amplicons in equal amounts and sequenced on an Illumina MiSeq machine (600V3, paired end).

Resulting reads were merged using USEARCH. Since several amplicons were longer than the sequenced range, these reads were concatenated. Further downstream processing was performed as described for bacterial sequencing, although error rates > 3 were excluded. Taxonomy was determined using the Bayesian classifier^46^ and the UNITE database^47^.

### Archaeal 16S rRNA gene sequencing

Abundance of archaeal reads was determined and analyzed as previously described^48^. In brief, archaeal signatures were amplified using the nested primer combination 344F-1041 and 519F-806R. Amplicons were sequenced at the Center for Medical Research, Medical University of Graz as described earlier^49^. The obtained reads were merged with USEARCH. ASV abundance was determined as described for the bacterial amplicons. Reads were classified using Bayesian classifier^46^ and the SILVA V132^45^ database. Bacterial ASVs were removed from the count table.

### Microbiota evaluation and statistical interpretation

Sequencing data was analyzed and visualized using R (v3.6.1). Data were compiled in a phyloseq^50^ (v.1.30.0) object. Taxonomic analysis was performed on total counts, while for α- and β-diversity counts were rarefied without replacement to 35 k reads for 16S or 10 k reads for ITS1. For fungal richness and diversity analysis, three sample pairs were excluded during rarefaction due to insufficient reads. α-Diversity metrics were determined using phyloseq and Picante^51^ (v1.8). All microbiome graphs were made with ggplot2 (v3.2.1). Differentially abundant ASVs were determined using DESeq2^52^ (v3.8) where a False Discovery Rate (FDR) below 0.05 was considered significant. Linear mixed effect models (lme4, v1.1–21) were used to test diversity metrics and differential abundance of selected taxa and covariate interactions. To test significant effects of study outcome or treatment on bacterial and fungal community composition, a multi-level PCA (mPCA) was performed using the mixomics package^53^ (v3.9). Subject stratified F-statistic based permutation (1000x) MANOVA (package vegan^54^, v2.5-6) on the first 10 components was used for significance.

### Quantitative PCR determination of fungal load

Fungal DNA was quantified according to the FungiQuant quantitative Polymerase Chain Reaction (qPCR) method^18^. Bacterial DNA load was estimated as previously described with modifications^55^. Final primer concentration was 500 nM and SensiFAST No-ROX (GC Biotech, Alphen aan den Rijn, the Netherlands) was used on a CFX96 (Bio-Rad, Veenendaal, the Netherlands). Annealing temperatures were 60 ℃ for FungiQuant and 66 ℃ for bacterial DNA. Data were analyzed using CFX Maestro and LinReg software^56^. One non-improver and two improver sample pairs were excluded because signal trespassed lower detection level (defined as concentration control signal × 2).

### Culture and identification of fecal *C. albicans* strains

In sterile working conditions, scrapings from frozen fecal samples were resuspended in 500 µL sterile phosphate-buffered saline (PBS). After vortexing, 100 µL of each sample was spread onto Sabouraud Dextrose Agar (SDA, Sigma Aldrich) plate and Yeast Potato Dextrose agar (YPD, Sigma Aldrich), both supplemented with 0.05 g L^-1^ chloramphenicol. Plates were incubated aerobically at 37 °C for 24–48 h. A maximum of ten morphologically similar colonies were processed per individual for identification. Matrix-Assisted Laser Desorption-Ionization Time-of-Flight Mass Spectrometry (MALDI-TOF MS, Bruker Daltonics, Bremen, Germany) was used to identify strains by using the manufacturer’s protocol for extended direct transfer (eDT) and ethanol/formic acid extraction.

### Yeast DNA isolation and Amplified Fragment Length Polymorphisms fingerprint analysis

DNA isolation was performed by bead beating yeast cells in cetyltrimethylammonium bromide (CTAB) buffer (100 mM Tris–HCl pH 8.4, 1.4 M NaCl, 25 mM EDTA pH 8.0, 2% CTAB) followed by phenol/chlorophorm extraction, isopropanol precipitation, and RNase A treatment. DNA concentrations were quantified by Qubit fluorometric method. AFLP analysis was performed as previously described, the selective EcoRI and MseI primers used were adapted (5’-Flu-GACTGCGTACCAATTCAC-3’, 5'-GATGAGTCCTGACTAAC-3', respectively)^57^. Amplicons were analyzed on a 3730xl Genetic Analyzer (Applied Biosystems, ThermoFisher Scientific).

### *C. albicans* growth curve

Patient-derived *C. albicans* strains were grown in Yeast Nitrogen Base (YNB, Sigma Aldrich) with amino acids and 2% glucose overnight (37 ℃, 200 rpm). Yeasts were diluted to 110^5^ cells ml^-1^ and 250 μL was seeded in a 24 W culture plate (VWR). The plate was incubated in a pre-warmed (37 ℃) Clariostar plate reader (Isogen Life Science, De Meern, the Netherlands), with optical density (OD) measurements at λ = 600 nm, at intervals of 30 min. Shaking was set at continuous movement (300 rpm, 60 s on/off) and before each measurement (500 rpm, 30 s). OD values were blank corrected and displayed values were measured in triplicate.

### Characterization of *C. albicans* virulence factors

Yeast was cultured overnight (30 ℃, 200 rpm) in Yeast Nitrogen Base broth (Wickerham formula w/AA & w/AS, BD Difco, France) with 2% dextrose. Cultures were diluted to 1 × 10^8^ cells ml^-1^ in PBS (without Ca^2+^, Mg^2+^, pH 7.3–7.5, Accugene, Lonza, Belgium). 5μL cell suspension was spotted in triplicate onto different culture media for phospholipase^58^, lipase^59^, esterase^60^, or secreted aspartyl proteinase (Sap) activity^61^. Assay plates were incubated at 37 ℃ and assessed after five days, except for lipase (seven days). Activity was based on the Pz index, calculated as 1–(diameter colony/diameter precipitation zone). Each experiment was repeated three times.

### Adhesion of *C. albicans* to epithelial cells

HT-29 colon carcinoma cells were maintained in DMEM (Lonza) supplemented with 10% heat-inactivated Fetal Calf Serum (FCS)(Serana), 2 mM glutamine (Lonza), 100U penicillin and 100 μg streptomycin (Lonza) at 37 ℃ and 5% CO_2_. Cells routinely tested negative for mycoplasma contamination. Cells were seeded at 3 × 10^5^ cells ml^-1^ in a 48-well culture plate (VWR) 6 days prior to the assay. Confluent monolayers were washed with DMEM before exposing them to yeast. *C. albicans* was grown overnight in Sabouraud Dextrose Broth (Sigma)(37 ℃, 200 rpm). 25 × 10^6^ yeast cells ml^-1^ were then incubated for 30 min in 0.5 mg mL^-1^ calcofluor white stain (Merck, Amsterdam, the Netherlands) (37 ℃, 200 rpm), washed and resuspended at 8 × 10^6^ cells ml^-1^ in DMEM without phenol red (Lonza). Next, HT-29 cells were exposed to 4 × 10^5^ yeast cells in 200 μl for 1 h at 37 ℃. Non-adhered cells were washed away and fluorescence intensity was measured on a Clariostar plate-reader (λ_exc_ = 350/15 nm, λ_em_ = 433/20 nm). All signals were blank (i.e. only HT-29 cells) corrected, and adhesion is expressed relative to unwashed yeast-exposed cells: (intensity adhered cells/intensity control well)*100%.

### Morphogenesis and gene expression of *C. albicans*

Overnight cultures of *C. albicans* were prepared in Sabouraud Dextrose Broth (37 ℃, 200 rpm). Yeast-to-hyphal transition was induced using 10% heat-inactivated FCS (Serana) for 4 h. For microscopy, 1 × 10^5^ cells ml^-1^ were incubated in a 6-wells culture plate (VWR). Brightfield photomicrographs were taken using a DM8i microscope (Leica, Amsterdam, the Netherlands) using 20 × magnification.

For transcription analysis, 1 × 10^6^ cells ml^-1^ were incubated in a 6 W microplate (VWR). Yeast cells were collected in TriReagent (Sigma-Aldrich). Cells were lysed by bead beating in a FastPrep-24 (MP Biomedicals; 3 × 1 min, 5.5 ms^-1^) followed by chlorophorm extraction and isopropanol precipitation. RNA concentrations were determined using Nanodrop ND-1000. For first strand synthesis, 1.5 μg RNA was subjected to DNAse pre-treatment (Promega, Leiden, The Netherlands) treatment before commencing first strand synthesis by random hexamers (2.5 μg mL^-1^, Promega), oligo dTs (5 μM, Sigma-Aldrich), dNTPs (0.5 mM), reverse transcriptase buffer, 20U RevertAid RT, 100U RiboLock (all Thermofisher Scientific). Gene expression was determined by qPCR on a LightCycler 480 II system (Roche Diagnostics, Almere, The Netherlands). Most stable reference genes were determined by GeNorm analysis and Linear Regression analysis^56,62,63^. All primers (Merck) were used at 500 nM (Supplementary Table 1). Relative expression is determined based on the geometric mean of two reference genes (*TDH3*, *PMA1*), and fold induction was calculated relative to unstimulated samples. Data points were omitted if no amplification was observed or if the Ct value was within 7 cycles of any control sample.

### Statistical analysis

Bioinformatics analysis was performed as described above. Baseline characteristics of both patient cohorts were not tested for significance in accordance with the STROBE guidelines^64^. Data of in vitro experiments was considered non-parametrically distributed and are displayed as median with separate data points. Data were tested using Kruskal–Wallis (KW) tests and Dunn’s post-hoc test in case of significant KW results using GraphPad Prism. Reported P-values are the result of general KW unless specified otherwise. P-values below 0.05 are considered statistically significant.

## Supplementary Information

Supplementary Information.

## Data Availability

All raw sequencing data have been submitted to the ENA database under accession number PRJEB44225.
