# Supplementary material for: Genetic and phenotypic diversity of fecal Candida albicans strains in irritable bowel syndrome
Source: Sci Rep. 2022 Mar 30;12:5391. doi: 10.1038/s41598-022-09436-x (PMC8967921; doi:10.1038/s41598-022-09436-x)
Supplement: Supplementary file 1 — Supplementary Information. [file 41598_2022_9436_MOESM1_ESM.pdf]

This document includes Supplementary Information

(Supplementary Table 1 & Supplementary Figures 1-5) to:

Genetic and phenotypic diversity of fecal *Candida albicans* strains in Irritable Bowel Syndrome

Isabelle A.M. van Thiel[1,2], Aimilia Stavrou[3,4], Auke de Jong[3,5], Bart Theelen[3], Mark Davids[6,7], Theodorus B.M. Hakvoort[1,2,7] Iris Admiraal-van den Berg[1,2,7], Isabelle C.M. Weert[1], Martine A.M. Hesselink-van de Kruijs[8], Duong Vu[3], Christine Moissl-Eichinger[9], Sigrid E.M. Heinsbroek[1,2,10], Daisy M.A.E. Jonkers[8], Ferry Hagen[3,5] Teun Boekhout[3,4], Wouter J. de Jonge[1,2,10,11], René M. van den Wijngaard[1,2,10,*]

1. Tytgat Institute for Liver and Intestinal Research, Amsterdam University Medical Centers, location AMC. Amsterdam, The Netherlands.
2. Amsterdam Gastroenterology Endocrinology Metabolism, Amsterdam UMC, location AMC. Amsterdam, The Netherlands.
3. Westerdijk Fungal Biodiversity Institute. Utrecht, The Netherlands.
4. Institute of Biodiversity and Ecosystem Dynamics (IBED), University of Amsterdam. Amsterdam, The Netherlands.
5. Department of Medical Microbiology, University Medical Center Utrecht. Utrecht, The Netherlands.
6. Laboratory of Experimental Vascular Medicine, Amsterdam University Medical Centers, location AMC. Amsterdam, The Netherlands.
7. Microbiota Center Amsterdam, Amsterdam University Medical Centers, location AMC. Amsterdam, The Netherlands.
8. Division Gastroenterology-Hepatology, Department of Internal Medicine, NUTRIM School of Nutrition and Translational Research in Metabolism, Maastricht University Medical Center+. Maastricht, The Netherlands.
9. Diagnostic and Research Institute of Hygiene, Microbiology and Environmental Medicine, Center for Microbiome Research, Medical University Graz. Graz, Austria.
10. Department of Gastroenterology and Hepatology, Amsterdam University Medical Centers, location AMC. Amsterdam, The Netherlands.
11. Department of General, Visceral, Thoracic and Vascular Surgery, University Hospital Bonn. Bonn, Germany.

Supplementary Table 1.
Primer sequences for quantitative PCR experiments.

Gene of interest	Identifier	Forward/Reverse	Primer sequences (5'-3')
TDH3 reference	XM_714816.1	Forward	CGGTCCATCCCACAAGGA
		Reverse	AGTGGAAGATGGGATAATGTTACCA
PMA1 reference	XM_712666.2	Forward	TTGCTTATGATAATGCTCCATACGA
		Reverse	TACCCCACAATCTTGGCAAGT
ALS1	XM_712984.2	Forward	CAACAGGCACCTCAGCATCTAC
		Reverse	CTCCACCAGTAACAGATCCACTAGTAA
ALS3	XM_705343.2	Forward	CAACTTGGGTTATTGAAACAAAAACA
		Reverse	AGAAACAGAAACCCAAGAACAACCT
NRG1	XM_710106.1	Forward	ACATGTGAAGCCCGTTTTGC
		Reverse	CTGTGTTGTTGTCTGTTGCGT
HGC1	XM_713897.2	Forward	GTCAGCTTCCTGCACCTCAT
		Reverse	TTGGTGGTGGTGGTTGCATA
SAP2	XM_705955.2	Forward	CCAATGAAGCCGGTGGTAGT
		Reverse	TATTTGTCCCGTGGCAGCAT
SAP4	XM_712961.1	Forward	GCCGATGGTTCTGTTGCAC
		Reverse	ACACCACCAATACCAACGGT
ECE1	XM_706502.1	Forward	CCATCATCCACCATGCTCCA
		Reverse	ACAGTAGGTGCTTGGTCAGC
CDR1	XM_718116.2	Forward	CCATGACTCCTGCTACCGTG
		Reverse	CCATCGAGACCAACCCAACA
MDR1	XM_714072.2	Forward	ACAATTTGGCCACCCCTGAA
		Reverse	CCAACACGGAACTACCCCAA

a

c

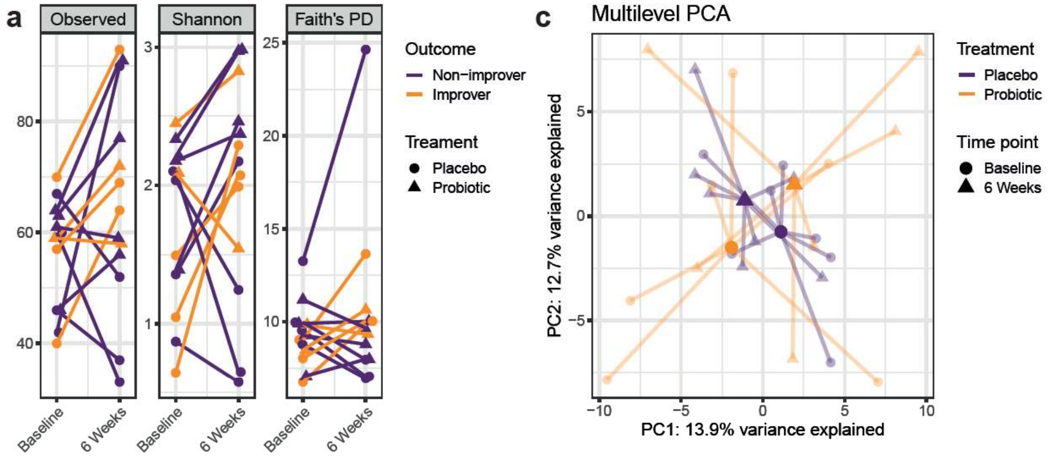

b

	Observed		Shannon		Faith's PD	
	**F value**	**P value**	**F value**	**P value**	**F value**	**P value**
Time_Point	2.359	0.159	2.33	0.161	1.19	0.304
Treatment	0.323	0.584	13.061	**0.006**	0.222	0.648
Effect	0.485	0.504	0.031	0.865	0.089	0.772
Time_Point:Treatment	0.001	0.979	0.003	0.958	0.702	0.424
Time_Point:Effect	0.343	0.573	0.854	0.38	0.425	0.531
Treatment:Effect	1.169	0.308	0.6	0.458	0.234	0.64
Time_Point:Treatment:Effect	1.215	0.299	8.013	**0.02**	0.04	0.847

Supplementary Figure 1. Mycobiota composition as expressed by Shannon index associates with probiotic treatment and outcome, although no community-based associated was determined. a-b) Alpha diversity as expressed by observed species, Shannon index, and Faith's Phylogenetic Distance of mycobiota samples. Shannon index is significantly different for allocated treatment. Results linear mixed effect models presented in b). c) Mycobiota composition, displayed in a multilevel principle component analysis, is not significantly different after treatment.

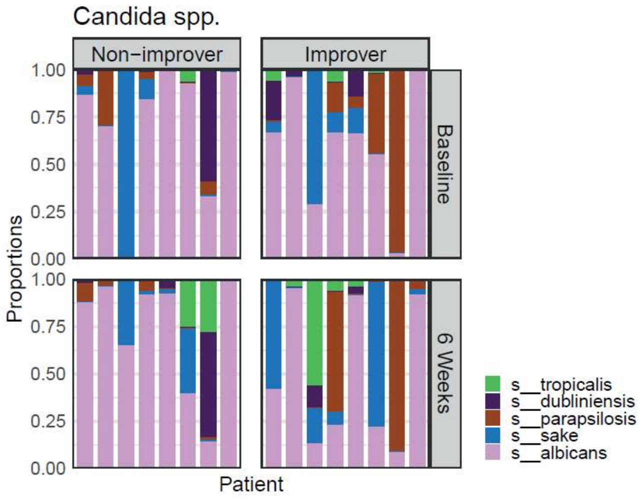

Supplementary Figure 2. Taxonomic analysis of *Candida* spp. reveals *Candida albicans* as most abundant species. Relative abundance of *Candida spp.* in each sample. Each column represents one patient. A minimal abundance threshold of 0.1% was used to remove low-confidence species for enhanced visualization purposes. *C. albicans* is most abundant between all samples, followed by *C. sake* and *C. parapsilosis.*

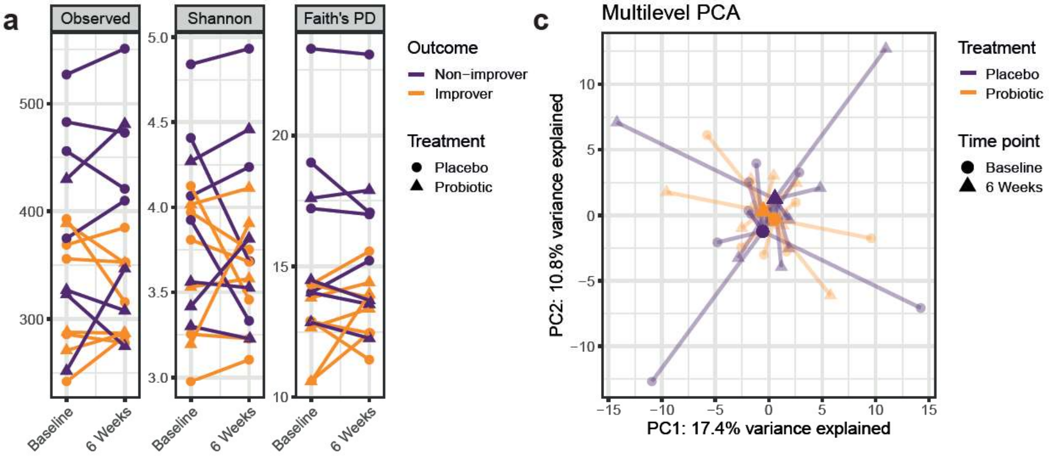

b

	Observed		Shannon		Faith's PD	
	**F value**	**P value**	**F value**	**P value**	**F value**	**P value**
Time_Point	0.045	0.835	0.014	0.906	0.681	0.425
Treatment	4.674	0.052	0.451	0.515	2.412	0.146
Effect	7.312	**0.019**	2.001	0.183	7.87	**0.016**
Time_Point:Treatment	0.103	0.754	5.958	**0.031**	1.131	0.309
Time_Point:Effect	0.619	0.447	0.5	0.493	3.59	0.082
Treatment:Effect	2.928	0.113	2.357	0.151	2.542	0.137
Time_Point:Treatment:Effect	0.143	0.712	0.058	0.813	1.479	0.247

Supplementary Figure 3. Bacterial microbiome composition is associated with treatment allocation to a limited extent. a-b) Alpha diversity metrics (Observed species, Shannon index, Faith's Phylogenetic distance) as shown for both outcome (improver/non-improver) and allocated treatment (placebo/probiotic). Shannon diversity generally associates with allocated treatment (p=0.031, Linear Mixed Effect Models (LME). Results linear mixed effects model presented in b). c) Community analysis by multilevel PCA based on Bray-Curtis dissimilarities. Symbol color represents allocated treatment. Composition is associated with treatment (p=0.007, PERMANOVA).

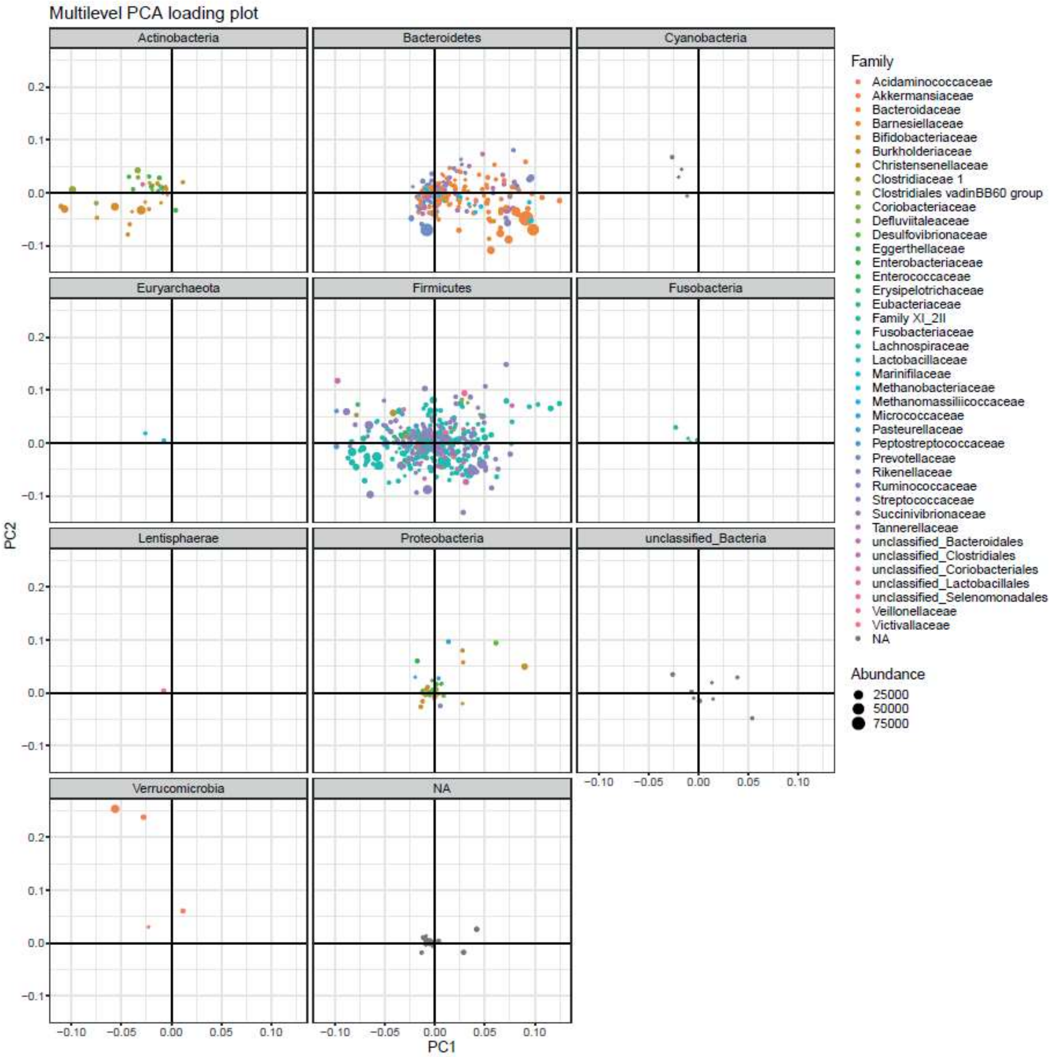

Supplementary Figure 4. MixOmics loading plot indicates shift of Verrucomicrobia. Loading plot indicating separation of microbial taxa along principal components (PC) 1 and PC2. Verrucomicrobia locate in the upper-left quadrant while greatest separation for other taxa is seen along PC1.

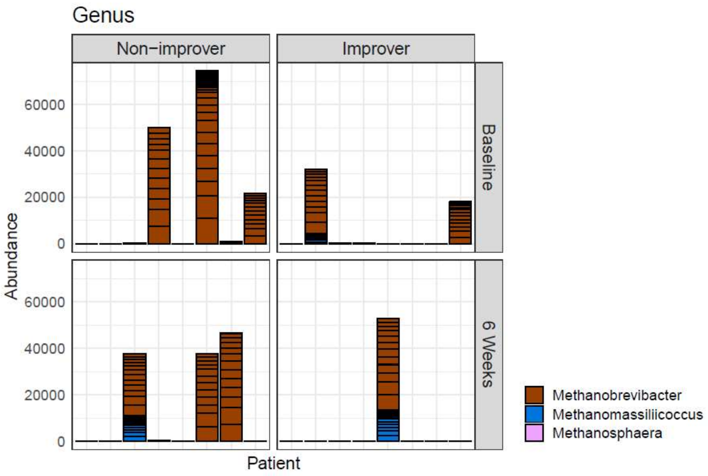

Supplementary Figure 5. Archaeal reads show high inter-individual variability. Archaeal reads counts displayed on genus level. Each column represents one paired sample. Each separated segment indicates individual species. Reads are highly variable between and within patients.
